# Identification of the Gene Expression Rules That Define the Subtypes in Glioma

**DOI:** 10.3390/jcm7100350

**Published:** 2018-10-13

**Authors:** Yu-Dong Cai, Shiqi Zhang, Yu-Hang Zhang, Xiaoyong Pan, KaiYan Feng, Lei Chen, Tao Huang, Xiangyin Kong

**Affiliations:** 1 School of Life Sciences, Shanghai University, Shanghai 200444, China; caiyudong@staff.shu.edu.cn (Y.-D.C.); shiqi.zhang@sund.ku.dk (S.Z.); 2 Department of Biostatistics, University of Copenhagen, Copenhagen 2099, Denmark; 3 Institute of Health Sciences, Shanghai Institutes for Biological Sciences, Chinese Academy of Sciences, Shanghai 200031, China; yhzhang@sibs.ac.cn (Y.-H.Z.); huangtao@sibs.ac.cn (T.H.); 4 Department of Medical Informatics, Erasmus Medical Centre, Rotterdam 3014ZK, The Netherlands; x.pan@erasmusmc.nl; 5 Department of Computer Science, Guangdong AIB Polytechnic, Guangzhou, 510507, China; kyfeng@gdaib.edu.cn; 6 College of Information Engineering, Shanghai Maritime University, Shanghai 201306, China; lchen@shmtu.edu.cn; 7 Shanghai Key Laboratory of Pure Mathematics and Mathematical Practice (PMMP), East China Normal University, Shanghai 200241, China

**Keywords:** glioma, gene expression, Monte Carlo feature selection, Johnson reducer algorithm, support vector machine

## Abstract

As a common brain cancer derived from glial cells, gliomas have three subtypes: glioblastoma, diffuse astrocytoma, and anaplastic astrocytoma. The subtypes have distinctive clinical features but are closely related to each other. A glioblastoma can be derived from the early stage of diffuse astrocytoma, which can be transformed into anaplastic astrocytoma. Due to the complexity of these dynamic processes, single-cell gene expression profiles are extremely helpful to understand what defines these subtypes. We analyzed the single-cell gene expression profiles of 5057 cells of anaplastic astrocytoma tissues, 261 cells of diffuse astrocytoma tissues, and 1023 cells of glioblastoma tissues with advanced machine learning methods. In detail, a powerful feature selection method, Monte Carlo feature selection (MCFS) method, was adopted to analyze the gene expression profiles of cells, resulting in a feature list. Then, the incremental feature selection (IFS) method was applied to the obtained feature list, with the help of support vector machine (SVM), to extract key features (genes) and construct an optimal SVM classifier. Several key biomarker genes, such as *IGFBP2*, *IGF2BP3*, *PRDX1*, *NOV*, *NEFL*, *HOXA10*, *GNG12*, *SPRY4*, and *BCL11A*, were identified. In addition, the underlying rules of classifying the three subtypes were produced by Johnson reducer algorithm. We found that in diffuse astrocytoma, PRDX1 is highly expressed, and in glioblastoma, the expression level of *PRDX1* is low. These rules revealed the difference among the three subtypes, and how they are formed and transformed. These genes are not only biomarkers for glioma subtypes, but also drug targets that may switch the clinical features or even reverse the tumor progression.

## 1. Introduction

Glioma is a general term describing a specific subgroup of brain cancers derived from glial cells [1]. Glial cells, which include oligodendrocytes [2], astrocytes [3], ependymal cells [4], and microglia [5], participate in the maintenance of the nerve microenvironment in the central and peripheral nervous systems. Due to the complicated cellular components of glial cells, tumors derived from such a group of nerve system cells with a general name, glioma, can be further clustered into various functional subgroups; moreover, each functional group may be originally derived from a unique functional subgroup [6,7]. Clinically, four common subgroups of glial malignancies with clear cell origins exist, namely, astrocytoma, oligodendroglioma, microglioma, and ependymal tumor, which are derived from astrocytes, oligodendrocytes, microglia cells, and ependymal cells, respectively [8,9].

Glioblastoma and astrocytoma are the two major subtypes of glioma with distinctive and typical clinical indications and genetic backgrounds [10]. Glioblastoma, in particular, has emerged to be one of the most aggressive cancers originating from the brain and has unknown cellular origins [11,12]. Clinically, in the early stage, glioblastoma is difficult to diagnose, due to its non-specific clinical features and its rapidly worsening symptoms [13]. One of the most significant diagnoses on glioblastoma is the recognition and distinction of primary glioblastoma from the secondary ones, due to their distinct pathological characteristics [14]. However, distinguishing the two pathological groups using only traditional clinical testing methods, including Magnetic Resonance Imaging (MRI), is challenging [14]. Under such circumstances, the genetic background of such subgroup of glioblastomas has been introduced to perform differential diagnosis. A specific biomarker in glioma, Isocitrate Dehydrogenase (NADP(+)) 1 *(IDH1)*, is found in more than 80% of secondary glioblastomas and only 5% of primary glioblastoma, implying that, at least in some conditions, genetic background (e.g., tumor malignancy indicator and *IDH1*) may be an optimal biomarker for the recognition of certain glioma subtypes [15,16]. On the other hand, astrocytoma can be further divided into at least two subgroups: diffuse astrocytoma and anaplastic astrocytoma [17]. Diffuse astrocytoma, also called low-grade or fibrillary astrocytoma, is a group of primarily slow-growing brain tumors specifically originating from astrocytes, and is different from glioblastoma on the level of cell origin and malignancy grade [18]. Furthermore, the anaplastic astrocytoma, derived from the pathological astrocytes, is a group of high grade (WHO level III/IV) undifferentiated gliomas with poor clinical prognosis [19]. Based on the genetic background of astrocytoma, mutations in gene *IDH1*, and specific copy number alterations in the genome, are two of the major molecular characteristics of astrocytoma [17].

Clinically, glioblastoma, diffuse astrocytoma, and anaplastic astrocytoma are the three different glioma subtypes with distinctive clinical features and respective genetic backgrounds [10]. However, glioblastoma can be derived from the early stage of diffuse astrocytoma, and the transition from diffuse astrocytoma to anaplastic astrocytoma is generally varied; therefore, distinguishing the three subgroups of gliomas, solely by means of their clinical features and identified genetic background, is difficult. Therefore, for the early classification and diagnosis of such gliomas, the detailed potential genetic diversity of gliomas should be further identified, and novel diagnostic criteria based on genetic biomarkers should be formulated. Traditionally, the identification of differentially expressed genes/biomarkers in different tumor subtypes generally rely on the bulk sequencing on the whole cell population with multiple cell subgroups. Therefore, some potential biomarkers, and differentially expressed genes in only one or two particular pathological cellular components, may be floated and missed [20]. Here, based on two specific single-cell sequencing results on the three subgroups of gliomas (glioblastoma, diffuse astrocytoma, and anaplastic astrocytoma) with confirmed mutant *IDH1* [21], we used several advanced computational methods to identify potential differentially expressed biomarkers for the distinction of the different glioma subgroups. The Monte Carlo feature selection (MCFS) [22] method was employed to analyze the gene expression profile of cells in three subgroups of gliomas. A feature list was produced, which was further used in the incremental feature selection (IFS) [23] method to extract key distinctive genes that contribute to the recognition of each glioma subtype, with the help of support vector machine (SVM) [24]. Several key biomarker genes, such as *IGFBP2*, *IGF2BP3*, *PRDX1*, *NOV*, *NEFL*, *HOXA10*, *GNG12*, *SPRY4*, and *BCL11A*, were analyzed and an optimal SVM classifier was constructed. In addition, we set up a series of rules via Johnson reducer algorithm [25] for the accurate distinction of the three glioma subgroups with vague pathological and genetic boundaries.

## 2. Materials and Methods

**Figure 1:**
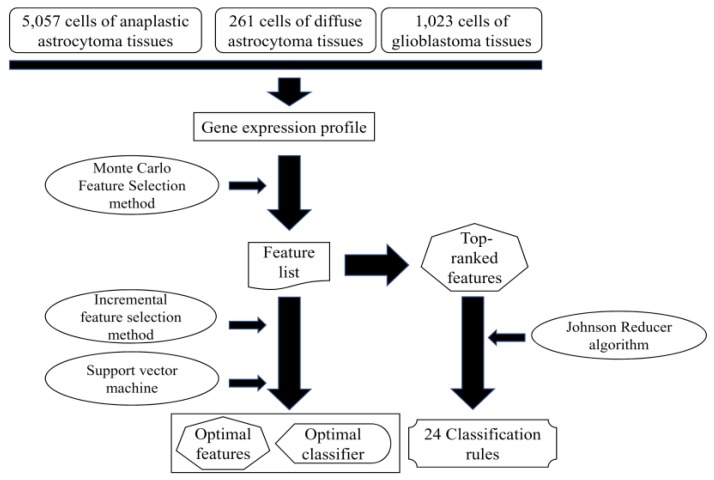
A flowchart to show the procedures of the method. The gene expression profile was analyzed by the Monte Carlo feature selection method, yielding a feature list. Some top-ranked features were used to produce classification rules via Johnson reducer algorithm. The incremental feature selection method used the feature list to extract optimal features and construct the optimal classifier, with the help of support vector machine.

In this study, we analyzed the single-cell expression profiles of glioma tissues from the dataset Gene Expression Omnibus (GEO) using machine learning methods. Based on the expression profiles, we identified the discriminative genes for different glioma subtypes by applying several feature selection methods and integrating with a support vector machine [24]. The detailed procedures are illustrated in Figure 1.

### 2.1. Dataset

We downloaded the processed single-cell gene expression profiles of 5057 cells of anaplastic astrocytoma tissues, 261 cells of diffuse astrocytoma tissues, and 1023 cells of glioblastoma tissues from GEO with accession number GSE89567 [21]. Venteicher et al. [21] disaggregated the tumor tissues into single cells and profiled them with Smart-seq2. They processed the single cell sequencing data with the following procedures: first, the reads were mapped to the human transcriptome with Bowtie; then, the expression values were estimated as transcripts per million (TPM) with RNA-Seq by Expectation Maximization (RSEM). Only the cells with more than 3000 expressed genes and with average housekeeping expression greater than 2.5 were included. The processed expression matrix with the TPM expression values of 23,686 genes in 5057 cells of anaplastic astrocytoma tissues, 261 cells of diffuse astrocytoma tissues, and 1023 cells of glioblastoma tissues were used to classify the cells from different disease tissues.

### 2.2. Feature Selection

In this study, we first used the MCFS [22] method to select informative genes, which can be used to classify different brain cancer subtypes and identify interpretable rules. Then, two-stage incremental feature selection (IFS) [23] was further employed based on the ranked features to refine the final “optimal” genes with strong discriminative power for the different subtypes of glioma.

#### 2.2.1. Monte Carlo Feature Selection Method

MCFS [22,26,27] is based on the extensively used decision tree and it adopts bootstrap sampling to rank information features for supervised classifiers. The general idea of MCFS is to randomly select several subsets from the original M features, in which each subset includes randomly selected m features (m ≪ M). Multiple decision trees are generated and evaluated on a bootstrapping dataset from the original training set. Here, the number of generated decision trees is denoted as *p*. The above process is repeated *t* times to obtain t feature subsets and *p* × *t* decision trees.

The relative importance (RI) is defined as a score of a feature involved in growing the *p* × *t* decision trees. The RI score of feature *g* can be calculated as follows:

(1) $$RI_{g}=\sum_{\tau =1}^{pt}{\left(wAcc\right)}^{u}IG(n_{g}(\tau )){\left(\frac{no.in\;n_{g}(\tau )}{no.in\;\tau }\right)}^{v},$$

where *wAcc* is the weighted accuracy, which is calculated as the mean accuracy of all classes; $n_{g}(\tau )$ indicates a node using feature *g* in decision tree τ; $IG(n_{g}(\tau ))$ is the information gain of $n_{g}(\tau )$; $no.in\;n_{g}(\tau )$ is the number of training samples in $n_{g}(\tau )$; no.in τ is the number of samples in decision tree τ; and *u* and *v* are two weighting factors, which were all set to 1, their default setting. After the RI score of each feature has been calculated, all features are ranked in a feature list according the descending order of their RI values. For formulation, this feature list was formulated as

(2) $$F=[f_{1},f_{2},\ldots ,f_{N}],$$

where *N* is the total number of features.

In this study, we used MCFS software package (Version 1.2.14) [28] to rank all 23,686 genes involved.

#### 2.2.2. Rule Learning

Based on the ranked genes from MCFS, we identified simple and interpretable rules for classifying different glioma subtypes using a rough set-based rule-learning algorithm. We detected interactions among the different genes that were represented as rules. A rule describes a relation between conditions (the left-hand-side of the rule) and the outcome (the right-hand-side). For example, a rule can be presented as an IF–THEN relationship based on expression values: IF Gene1 ≥ 5.1 AND Gene2 ≤ 8.9, THEN subtype = “glioblastoma”. We identified the rules using the Johnson reducer algorithm [25] implemented in the MCFS software package.

#### 2.2.3. Incremental Feature Selection

Incremental feature selection (IFS) [23] is an ideal method used to screen a set of optimal features to accurately distinguish samples from different groups. Here, IFS was executed on the feature list *F*, in which features are ranked in descending order of their RI values. Clearly, features with high ranks were important and positive for classification. Thus, combining some top features can help a classification algorithm (e.g., SVM) produce good performance. There were 23,686 features in the feature list, inducing lots of time to test all possible feature subsets. In view of this, we designed a two-stage IFS method. 

In the first stage, we used a large step of 10 to generate several feature subsets, denoted as $F_{1}^{1},F_{2}^{1},\ldots ,F_{m}^{1}$, where the *i*-th feature subset included top *i* × 10 features in *F*, that is, $F_{i}^{1}=[f_{1},f_{2},\ldots ,f_{i\times 10}]$. In other words, we constructed a series of feature subsets that contained first ten, twenty, thirty, and so forth, features in the feature list *F*. Then, for each of these feature subsets, all cells were represented by features in this set, and SVM was executed on these cell representations, evaluated by ten-fold cross-validation. After testing all these feature subsets, we can determine the feature subset that can help SVM provide good performance, thereby obtaining a feature interval [min, max]. Clearly, this interval should contain the size of feature subset that can yield the best performance for SVM.

In the second stage, we further constructed a series of feature subsets based on the interval [min, max] obtained in the first stage. In detail, feature subsets, denoted as $F_{\min}^{2},F_{\min+1}^{2},\ldots ,F_{\max}^{2}$, were generated, where $F_{i}^{2}\;(\min\leq i\leq \max)$ contained the first *i* features in feature list *F*. For example, if min = 300 and max = 600, the second stage of IFS method constructed the feature subsets containing first 300–600 features in the feature list *F*. It is clear that we did careful searching at this stage to find a better feature subset, which may not be tested in the first stage. Similarly, the SVM was executed on cells that were represented by features in each of these feature subsets, also evaluated by ten-fold cross-validation. According to the predicted results, the feature subset producing the best performance for SVM can be extracted. The features in this subset were considered as optimal features, and a corresponding optimal classifier was built on these optimal features.

### 2.3. Support Vector Machine

SVM [24] is a widely used supervised-learning algorithm based on the statistical learning theory, which is applied to handle many biological problems [29,30,31,32,33,34,35,36,37]. SVM performs linear classification and non-linear classification problems. The basic principle is to infer a hyperplane with a maximum margin between two classes of samples. The larger the margin is, the lower the generalization error becomes. The SVM first maps the data into high-dimensional linear space via kernel trick, such as Gaussian kernel; then, it fits the linear function in a high-dimensional space. Mainly developed for binary class problems, SVM can be extended for multi-class problems. For multi-class classification, SVM adopts “One Versus the Rest” strategy. Hence, to acquire m-class classifiers, SVM constructs a set of binary classifiers $svm_{1},svm_{2},\ldots ,svm_{m}$, in which each is trained to separate one class from the rest.

In this study, we used the tool “SMO” in Weka (version 3.8.0), which implements one type of SVMs that is optimized by sequential minimum optimization (SMO) [38]. For convenience, this tool was executed with its default parameters. In detail, the kernel was polynomial function and the tolerance parameter was 0.001. The Weka software can be downloaded at a public URL [39]. 

### 2.4. Performance Measurement

In this study, we considered cells in three glioma tissues. As mentioned in Section 2.1, the anaplastic astrocytoma tissues contained most cells (5057), while diffuse astrocytoma tissues contained least cells (261), meaning it is an imbalanced dataset. For this type of dataset, the overall accuracy cannot correctly indicate the quality of predicted results because it is highly related to the accuracy of the largest class. For binary classification, Matthews correlation coefficient (MCC) [40,41,42,43] is regarded as a balanced measure, even if the classes are of very different sizes. In this study, we employed its multiclass version [44], which was proposed by Gorodkin, to evaluate the prediction performance using ten-fold cross-validation [31,45,46,47]. It is believed that it can evaluate the performance of classifiers in a fair circumstance. Its brief description is as below.

For example, *N* samples (*i* = 1, 2, …, *N*) and *C* classes (*j* = 1, 2, …, *C*) are formulated. Let $X={\left(x_{ij}\right)}_{N\times C}$ be a matrix representing the predicted classes of samples, and $x_{ij}\in \{0,1\}$ is a binary output variable; $x_{ij}$ equals to 1 if the sample *i* is predicted to be class *j*; otherwise, $x_{ij}$ is 0. The matrix $Y={\left(y_{ij}\right)}_{N\times C}$ is defined as another matrix indicating the true classes of samples, where the binary variable $y_{ij}=1$ when the sample *i* belongs to class *j*; otherwise, it is set to 0.

The MCC can be defined as a discretization of the correlation for binary variables, which is specified by

(3) $$MCC=\frac{\mathrm{cov}(X,Y)}{\sqrt{\mathrm{cov}(X,X)\mathrm{cov}(Y,Y)}}=\frac{\sum_{i=1}^{n}\sum_{j=1}^{C}\left(x_{ij}-{\bar{x}}_{j})(y_{ij}-{\bar{y}}_{j}\right)}{\sqrt{\sum_{i=1}^{n}\sum_{j=1}^{C}{\left(x_{ij}-{\bar{x}}_{j}\right)}^{2}\sum_{i=1}^{n}\sum_{j=1}^{C}{\left(y_{ij}-{\bar{y}}_{j}\right)}^{2}}},$$

where ${\bar{x}}_{j}$ and ${\bar{y}}_{j}$ are the mean values of numbers of $x_{j}$ and $y_{j}$, respectively. The value of MCC ranges from −1 to 1; the higher the MCC value is, the better the performance the classifier achieves.

## 3. Results

**Figure 2:**
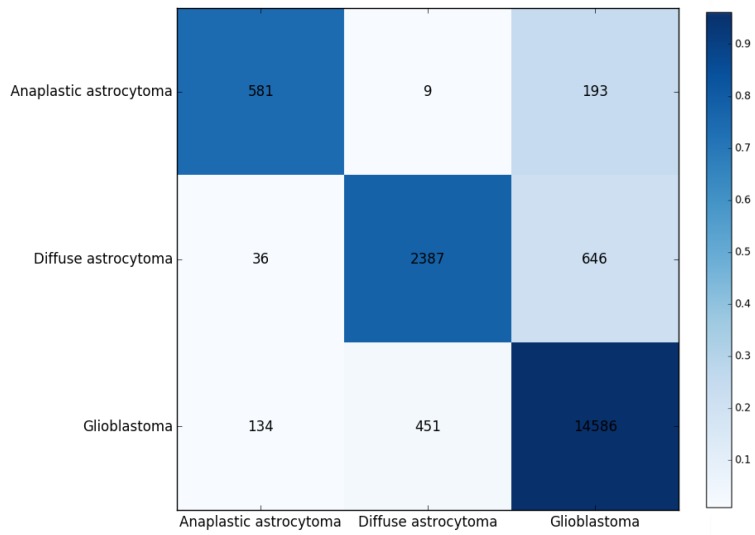
Confusion matrix for 10-fold cross-validation based on the detected 24 rules for classifying three glioma subtypes. The numbers were pooled from running 10-fold cross-validation on the training data thrice. The darker the color is, the higher the proportion is.

In this study, we first used MCFS to rank the genes for different glioma subtypes. The corresponding RI values of the 23,686 genes involved in this study, and the feature list *F* that was obtained by increasing order of features’ RI values, are provided in the Table S1. We further detected 24 rules (Table 1) based on some top-ranked genes from MCFS using Johnson reducer algorithm. More details about these rules are discussed in Section 4. Moreover, these rules are used to classify the three glioma subtypes (diffuse astrocytoma, glioblastoma, and anaplastic astrocytoma). We yielded a predicted accuracy 0.923, a weighted accuracy 0.827, and an MCC of 0.764 by considering the prevalence of different classes. The confusion map for ten-fold cross-validation was repeated three times, in which the rules were applied to classify glioma subtypes, as shown in Figure 2, where the numbers are pooled from running ten-fold cross-validation thrice.

**Figure 3:**
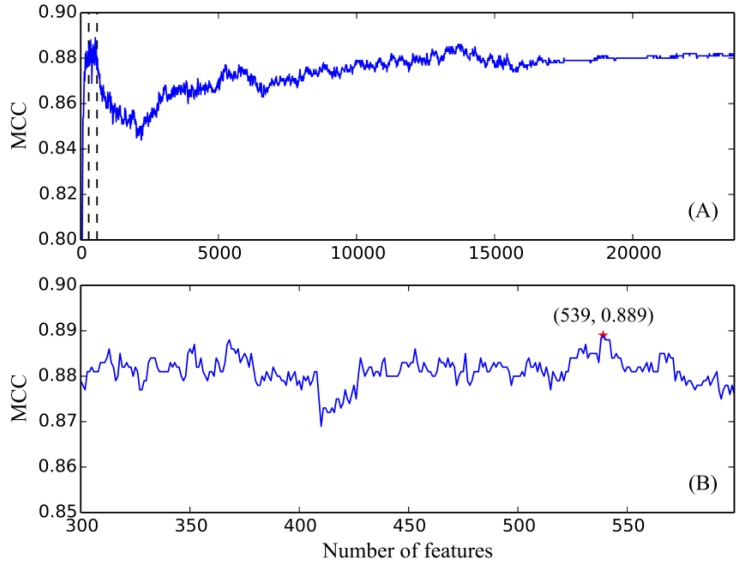
Incremental feature selection (IFS) curve derived from the IFS method and support vector machine (SVM) classifier. X-axis is the number of features involved in building classifiers. Y-axis is their corresponding *MCC* values. (**A**) IFS curve with X-values of 10 to 23,686. The selected feature intervals were 300 and 600, which were marked with two vertical lines; (**B**) IFS curve with X-values of 300 to 600 for the SVM classifier. When the 539 features were selected, the *MCC* value (0.889) is the highest.

We applied SVMs to classify different glioma subtypes using the selected features from two-stage IFS method. In the first stage of IFS method, a series of feature subsets with a step of 10, that is, a set of feature subsets containing first ten, twenty, thirty, and so forth, features in the feature list *F*, was constructed. We trained an SVM classifier on each of these feature subsets, which was evaluated using ten-fold cross-validation. We obtained the best MCC 0.888 using the first 540 features in *F*. Furthermore, the second highest MCC (0.886) was yielded by the first 370 features. In view of this, we determined the feature interval as [300, 600]. Then, we further constructed a second series of feature subsets with a step of one in the feature number interval [300, 600] in the second stage of IFS method, that is, we constructed the feature subsets containing first 300–600 features in *F*. Similarly, by testing on these feature subsets, we yielded the highest MCC 0.889 when the top 539 features were used to train the SVM classifier. Meanwhile, the predicted accuracy values for three glioma subtypes (diffuse astrocytoma, glioblastoma, and anaplastic astrocytoma) were 0.981, 0.969, and 0.871, respectively, and the overall accuracy was 0.963. Furthermore, we showed the trends of MCCs corresponding to the number of features involved in building the SVM classifiers (Figure 3). In Figure 3A, boundaries of feature interval are labeled with red markers. Figure 3B zooms in the curve between 300 and 600 on the X-axis, in which the optimal MCC value, 0.889, is marked with a red star. The predicted accuracies and MCCs in different feature subsets are listed in Table S2. In this study, we used several feature selection methods for constructing an SVM classifier. However, because we generated the feature list based on all samples before doing ten-fold cross-validation on different feature subsets, the information of testing samples was slightly included in the training procedure, which may enhance the performance of each classifier. Considering that the final SVM classifier gave good performance (MCC = 0.889), it is believed that the performance of the final SVM classifier would be still good if we did a stricter test.

## 4. Discussion

We presented a novel computational workflow for the identification of core distinctive expression patterns of the three glioma subtypes and summarized a series of quantitative rules for the accurate recognition of such subtypes. According to recent publications, all identified high-related distinctive expressed genes and quantitative rules can be verified. Due to the limitation of the article length, analyzing each identified gene and its corresponding rules is impossible. Therefore, we screened out the high-ranked genes and obtained their respective optimal rules for each glioma subtype to be used for further discussion. The detailed analysis can be seen below.

### 4.1. Analysis of Optimal Genes That May Contribute to the Recognition of Each Glioma Subtype

**Figure 4:**
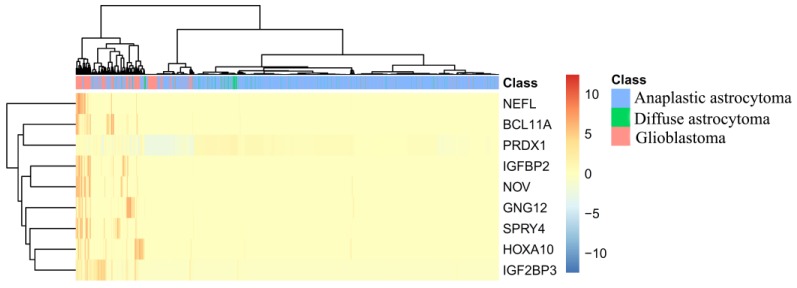
A heatmap to illustrate the expression level of three glioma subtypes on top nine genes.

In this section, we took top nine features (genes) in the feature list yielded by the MCFS method, which are listed in Table 2, for detailed analysis. To clearly display the expression level of three glioma subtypes on these genes, a heatmap was plotted in Figure 4. We can figure out that these genes can easily distinguish anaplastic astrocytoma and diffuse astrocytoma from glioblastoma. As for further distinction on anaplastic astrocytoma and diffuse astrocytoma, though two such groups of samples are mingled together, diffuse astrocytoma has specific and sporadic individual high expression level on one or more of such genes, while in anaplastic astrocytoma, almost all optimal genes were not detected. Therefore, from Figure 4, though according to the clustering results, samples of anaplastic astrocytoma and diffuse astrocytoma are mingled and, actually, the top nine genes can still contribute toward distinguishing samples in different types with unique expression pattern.

*IGFBP2*, as the top gene in the feature list yielded by MCFS method, encodes one of the six similar proteins that bind to insulin-like growth factors I and II (IGF-I and IGF-II) [48]. As for its differential expression pattern on the three glioma subtypes, *IGFBP2* has been confirmed to be highly expressed in gliomas with high malignancies, such as glioblastoma and anaplastic astrocytoma, but expressed low in the relatively binary astrocytoma, the diffuse astrocytoma [49,50]. Therefore, *IGFBP2* may be another potential biomarker for the distinction of the three glioma subtypes with positive IDH-1. Similarly, another insulin-like growth factor-binding protein encoded by *IGF2BP3* (rank 7) may also be an optimal differential marker for the identification of different glioma subtypes. The next gene, *PRDX1* (rank 2), encodes an antioxidant enzyme as a member of the peroxiredoxin family [51]. As for its expression pattern in different glioma subtypes, *PRDX1* may be connected to the poor prognosis of glioma subtypes, including glioblastoma and astrocytoma [52,53]. In addition, the expression pattern of *PRDX1* may be a potential biomarker for the recognition of astrocytoma in elderly patients, confirming its potential role in the differential diagnosis of glioma [53]. *NOV* (rank 3), encodes a small secreted cysteine-rich protein in the CCN family, and participates in fibrosis and cancer development-associated biological processes [54,55]. According to its distinctive pathological role in different glioma subtypes, *NOV* inhibits the proliferation and promotes the migration and invasion of the malignant cells in glioblastoma [56]. However, no direct reports have been presented to summarize the role of *NOV* in astrocytoma, implying the differential biological function and expression pattern of such gene in different glioma subtypes. The next gene, *NEFL* (rank 4), encodes a member of the neurofilaments and is involved in the maintenance of neuronal caliber [57]. *NEFL* (also known as NF68) has been functionally connected to a ligand of PPAR gamma PGJ2, and participates in the tumorigenesis of glioblastoma [58]. With the specific abnormal expression pattern of *NEFL*, glioblastoma, one of the glioma subtypes, can be accurately identified by such a gene.

The gene *HOXA10* (rank 5) is involved in a developmental regulatory system that provides cells with specific positional identities on the anterior–posterior axis as a member of transcription factors called homeobox genes [59,60]. The methylation and expression of *HOXA10* has been functionally connected to the stem cell pattern of glioma cells [61]. According to recent publications, the stem cell signature of diffuse astrocytoma is quite different from the other two glioma subtypes, indicating that *HOXA10* may be a potential biomarker for the identification of diffuse astrocytoma cells and validating the efficacy and accuracy of our prediction [62,63]. *GNG12* (rank 6), as another optimal biomarker, contributes to the distinction of different glioma subtypes. As a modulator and transducer in various transmembrane signaling system, such a gene is required for the guanosine triphosphatases (GTPase) activity, which participates in the replacement of guanosine diphosphate (GDP) by GTP [64]. GTPase-associated biological processes are related to specific tumor behavior, like migration, invasion, and proliferation, in multiple tumor subtypes, including glioma [65,66]. Considering that the fundamental tumor behavior of the three tumor subtypes are quite different [1,67], we speculate that one of the GTPase-associated regulators, *GNG12*, may have different expression pattern in glioma. The following two optimal genes, *SPRY4* (rank 8) and *BCL11A* (rank 9), act differentially on the three glioma subtypes according to recent publications. No direct evidence confirmed that *SPRY4* may act differentially in glioblastoma and the two astrocytomas. However, a recent study confirmed that, in gliomas, the expression pattern of *SPRY4* may be related to the cell proliferation, metastasis, and epithelial–mesenchymal transition processes [68]. Therefore, it is reasonable for us to speculate that *SPRY4* may have a differential expression pattern in such subtypes, and act as a potential biomarker based on its expression level [69,70]. *BCL11A*, encoding a C2H2 type zinc-finger protein, participates in brain development, leukemogenesis, and hematopoiesis [71,72]. Early in 2012, as a potential oncogene, *BCL11A* has been reported to contribute to glioblastoma with specific expression pattern [73]. However, no such report has confirmed the contribution of *BCL11A* on astrocytoma, validating that it may be a potential biomarker for the distinction of the three glioma subtypes.

To sum up, the top nine optimal genes have been confirmed to have specific expression patterns in the three candidate glioma subtypes, contributing to further subclassification by recent publications and validating the efficacy and accuracy of our study.

### 4.2. Analysis of Optimal Rules for Quantitative Identification of Each Glioma Subtype

Apart from potential biomarkers, we further set up a quantitative identification system involving 24 quantitative rules based on the expression level of each specific parameter (gene). According to recent publications, the tendency and specific threshold of each rule can be confirmed, proving the utility of these rules. Limited by the article length, we screened out the representative rules for the identification of each glioma subtype.

Ten rules were formulated to contribute to the identification of diffuse astrocytoma involving multiple functional genes. To validate the efficacy and accuracy of such rules, we summarized the expression pattern of various related sequencing datasets. Due to the limitation of article length, analyzing each rule individually is impossible. Therefore, we chose three optimal rules for further analysis: rule 3, rule 4, and rule 5. These three rules are involved in 9 genes, each sharing a high expression pattern of *XIST* with different thresholds. At relatively early stage of gliomas, the degree of malignancy is low in diffuse astrocytoma. *XIST*, as the shared gene, has been confirmed to participate in tumor-suppressive biological processes; its high expression corresponds with a specific pathological pattern [74]. The high expression of *XIST* has been shared by most of the diffuse astrocytoma, validating their efficacy and accuracy of such rules. Apart from *XIST*, the two homologues, namely, *RPL7* and *RPL8*, have also been predicted to have quantitative patterns in diffuse astrocytoma. Based on the rules, *RPL7* has a uniquely high expression pattern, while *RPL8* has a relatively low expression pattern. According to recent publications, such a pattern has been identified in the early stage of human fetal astrocytes [75]. Considering the similarity of fetal and tumor at the differential state level, we speculate that in the diffuse astrocytoma, the expression level of *RPL7* and *RPL8* may be quite different from the other two glioma subtypes [74]. Similarly, genes like *EGR1* [76], *EIF3C* [77], *HNRNPH1* [78], *C1orf61* [79], *CYP51A1* [80], and *CDR1* [81], have also been validated by recent publications.

Apart from such filtered rules that contribute to the identification of diffuse astrocytoma, thirteen rules are presented for the validation on glioblastoma. We screened out rule 11 and rule 12 for detailed analysis. Rule 11 involves four functional genes, indicating that high expression of *HSPA1B* and *MARCKS*, together with the low expression of *RPSAP58* and *PRDX1*, may indicate that a patient may suffer from glioblastoma. *HSPA1B* is highly expressed in glioblastoma and is related to the pharmacological effects of erlotinib [82]. Meanwhile, *MARCKS* is a prognosis reporter for glioblastoma and contributes to the intracranial tumor proliferation rate [83]. Therefore, as a malignant tumor subtype glioblastoma, the expression of *MARCKS* may be a potential biomarker for the identification of glioblastoma. The remaining two downregulated genes, *RPSAP58* and *PRDX1*, obtained similar evidences [10,52]. Likewise, in rule 12, three genes, including *COL20A1*, *CBR1*, and *MTRNR2L2*, are upregulated, and *TCF12* are downregulated [84,85]. Compared with the other two subtypes of astrocytoma, all these four genes have been confirmed, at the level of expression patterns, validating the high efficacy and accuracy of this rule. Samples that do not conform to any one of the rules are considered an anaplastic astrocytoma.

In conclusion, because of the limitation of the article’s length, we cannot analyze the rules individually. However, all rules can be validated by recent publications, implying the efficacy and accuracy of these quantitative rules. Therefore, based on the single-cell sequencing data, we tried to identify the core functional markers and set up the quantitative rules for such distinction. This study may not only screen out a group of candidate biomarkers for the recognition of different tumor subtypes, but also provide us a novel tool for the exploration and recognition of tumor-associated genes.

## Figures and Tables

**Table 1:** Twenty-four detected rules for classifying different glioma subtypes.

Rules	Criteria	Glioma Subtype	Rules	Criteria	Glioma Subtype
Rule1	XIST ≥ 2.725 LOC100190986 ≤ 1.956 GATM ≥ 4.826 PRDX1 ≥ 6.064	diffuse astrocytoma	Rule2	XIST ≥ 3.588 LOC100190986 ≤ 1.609 SLC1A3 ≥ 5.404 HLA-B ≤ 7.228	diffuse astrocytoma
Rule3	XIST ≥ 3.132 RPL7 ≥ 9.478 RPL8 ≤ 7.502 EGR1 ≤ 6.442	diffuse astrocytoma	Rule4	XIST ≥ 2.601 EIF3C ≤ 0.477 HNRNPH1 ≥ 6.813 C1orf61 ≤ 6.456	diffuse astrocytoma
Rule5	XIST ≥ 2.395 CYP51A1 ≥ 5.810 CDR1 ≥ 6.717	diffuse astrocytoma	Rule6	XIST ≥ 2.395 SKP1 ≥ 6.479 SEPT7 ≥ 5.342 RPL30 ≥ 7.419	diffuse astrocytoma
Rule7	XIST ≥ 2.395 SFPQ ≥ 4.772 JAM3 ≤ 0.000	diffuse astrocytoma	Rule8	XIST ≥ 3.021 RPL30 ≥ 8.453 PPIA ≥ 7.077 DDX5 ≤ 6.823	diffuse astrocytoma
Rule9	PCDHB7 ≥ 3.827 HNRNPH1 ≥ 6.670	diffuse astrocytoma	Rule10	RHOB ≥ 6.545 HSPA1A ≥ 4.446	diffuse astrocytoma
Rule11	RPSAP58 ≤ 1.280 HSPA1B ≥ 5.291 PRDX1 ≤ 0.000 MARCKS ≥ 3.464	glioblastoma	Rule12	TCF12 ≤ 4.952 COL20A1 ≥ 0.800 CBR1 ≥ 0.4222 MTRNR2L2 ≥ 12.850	glioblastoma
Rule13	NRCAM ≤ 0.999 HSPA1B ≥ 4.754 XIST ≥ 1.034 HSPA1B ≥ 7.275	glioblastoma	Rule14	RPSAP58 ≤ 1.414 PRDX1 ≤ 1.657 MTRNR2L8 ≥ 12.074 RPL8 ≥ 7.374	glioblastoma
Rule15	NRCAM ≤ 2.392 FOS ≤ 5.642 RPL35 ≥ 6.606 C1orf61 ≥ 6.700 MARCKS ≤ 4.770	glioblastoma	Rule16	FAM110B ≤ 2.527 RPSAP58 ≤ 0.165 NEAT1 ≥ 5.045 ITPR2 ≥ 2.118 HLA-C ≥ 6.293 NAPSB ≥ 4.988	glioblastoma
Rule17	FAM110B ≤ 2.607 RPSAP58 ≤ 0.000 SUSD5 ≥ 0.573 SUSD5 ≥ 2.515	glioblastoma	Rule18	TCF12 ≤ 4.215 RHOB ≤ 0.180 TMBIM6 ≤ 4.695 RPS26 ≤ 5.572 JAM3 ≥ 1.876	glioblastoma
Rule19	RIA2 ≤ 3.045 PRDX1 ≤ 0.000 MCL1 ≤ 2.387	glioblastoma	Rule20	NRCAM ≤ 1.090 DDX5 ≤ 6.520 SIRPB1 ≥ 1.014 EIF1 ≤ 7.690 NDUFA4 ≥ 0.811	glioblastoma
Rule21	SMOC1 ≤ 1.959 RPSAP58 ≤ 0.000 RPS26 ≤ 4.504 APOE ≤ 0.797 RPL7A ≥ 7.267	glioblastoma	Rule22	NRCAM ≤ 0.548 CD97 ≥ 0.856 CYBB ≥ 5.756 RPSAP58 ≤ 0.952 ITPR2 ≥ 2.769 EIF1 ≤ 8.648	glioblastoma
Rule23	NRCAM ≤ 0.548 MT2A ≥ 8.374 PFKFB3 ≥ 4.147	glioblastoma	Rule24	Other conditions	anaplastic astrocytoma

**Table 2:** Top nine genes yielded by Monte Carlo feature selection (MCFS) method.

Rank	Gene Symbol	Description	Relative importance (RI)
1	IGFBP2	Insulin-Like Growth Factor Binding Protein 2	0.1375
2	PRDX1	Peroxiredoxin 1	0.1226
3	NOV	Nephroblastoma Overexpressed	0.1194
4	NEFL	Neurofilament Light	0.1100
5	HOXA10	Homeobox A10	0.1059
6	GNG12	G Protein Subunit Gamma 12	0.0942
7	IGF2BP3	Insulin Like Growth Factor 2 MRNA Binding Protein 3	0.0891
8	SPRY4	Sprouty RTK Signaling Antagonist 4	0.0865
9	BCL11A	B Cell CLL/Lymphoma 11A	0.0847

## Supplementary Materials

The following are available online at http://www.mdpi.com/2077-0383/7/10/350/s1, Table S1: The involved 23,686 features are ranked by their RI values derived from MCFS method, Table S2: Corresponding accuracies of individual classes, overall accuracy, and MCCs using different number of features are selected by IFS method and SVM classifiers. Large materials used in this study, including original gene expression profiles and the final profiles on 539 genes that can be adopted to set up the optimal SVM classifier, can be accessed at https://cloud2010.github.io/.
